# Gene prioritization in Type 2 Diabetes using domain interactions and network analysis

**DOI:** 10.1186/1471-2164-11-84

**Published:** 2010-02-02

**Authors:** Amitabh Sharma, Sreenivas Chavali, Rubina Tabassum, Nikhil Tandon, Dwaipayan Bharadwaj

**Affiliations:** 1 Functional Genomics Unit, Institute of Genomics and Integrative Biology, CSIR, Delhi, India; 2 Department of Endocrinology, All India Institute of Medical Sciences, New Delhi, India

## Abstract

**Background:**

Identification of disease genes for Type 2 Diabetes (T2D) by traditional methods has yielded limited success. Based on our previous observation that T2D may result from disturbed protein-protein interactions affected through disrupting modular domain interactions, here we have designed an approach to rank the candidates in the T2D linked genomic regions as plausible disease genes.

**Results:**

Our approach integrates Weight value (Wv) method followed by prioritization using clustering coefficients derived from domain interaction network. Wv for each candidate is calculated based on the assumption that disease genes might be functionally related, mainly facilitated by interactions among domains of the interacting proteins. The benchmarking using a test dataset comprising of both known T2D genes and non-T2D genes revealed that Wv method had a sensitivity and specificity of 0.74 and 0.96 respectively with 9 fold enrichment. The candidate genes having a Wv > 0.5 were called High Weight Elements (HWEs). Further, we ranked HWEs by using the network property-the clustering coefficient (C_i_). Each HWE with a C_i _< 0.015 was prioritized as plausible disease candidates (HWEc) as previous studies indicate that disease genes tend to avoid dense clustering (with an average C_i _of 0.015). This method further prioritized the identified disease genes with a sensitivity of 0.32 and a specificity of 0.98 and enriched the candidate list by 6.8 fold. Thus, from the dataset of 4052 positional candidates the method ranked 435 to be most likely disease candidates. The gene ontology sharing for the candidates showed higher representation of metabolic and signaling processes. The approach also captured genes with unknown functions which were characterized by network motif analysis.

**Conclusions:**

Prioritization of positional candidates is essential for cost-effective and an expedited discovery of disease genes. Here, we demonstrate a novel approach for disease candidate prioritization from numerous loci linked to T2D.

## Background

Type 2 Diabetes (T2D) is a complex disease, encompassing various metabolic abnormalities influenced by both gene-environment and gene-gene interactions. Methodologies involving linkage analysis and/or association studies have been extensively exploited to identify the underlying genetic factors. Recent, advances using genome wide association (GWA) studies undertaken in large sample sets have provided a few susceptibility genes [1]. The success of GWA studies relies on the discovery of common variants of common diseases, however rare variants may also influence the risk of type 2 diabetes that are not captured in GWA studies. Hence, all these approaches have yielded very limited success, warranting new approaches, complementing the existing ones for disease gene discovery. Linkage analyses localize disease linked markers onto chromosomal regions that may correspond up to 30 Mb harboring several hundred genes [2]. Ideally, the information obtained through large number of genome-wide linkage studies has to be utilized to search T2D genes using a positional candidate based association studies. But, it is cost expensive and labor intensive apart from being time consuming to screen for each gene in the T2D linked region. Therefore, it is necessary to prioritize positional genes located in the linked chromosomal regions that would facilitate and expedite the identification of disease genes. Previously, a study used meta-analysis approach to prioritize disease genes from T2D and obesity linked regions based on consensus gene prioritization methods [3]. However, consensus methods are not always appropriate as they are primarily based on keyword similarity or phenotypes like biomedical text searches and associated pathological conditions for prediction [4]. Recently, protein-protein interaction data have also been extensively exploited for candidate gene prioritization in monogenetic as well as complex diseases [4-6].

**Figure 1:**
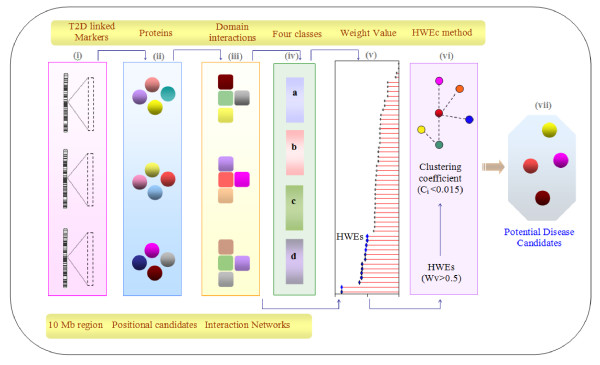
**Elucidation of the components used for the ranking the positional candidates in T2D**.

Previously, we demonstrated that T2D may result from disturbed protein-protein interactions resulting through disrupted modular domain interactions [7]. From this lead, here we have developed systems biology approach based on domain-domain interactions to prioritize positional candidates located in T2D linked regions (Fig.1). The method assumes that the functional relationship of protein-protein interactions is primarily facilitated by domains of the interacting proteins [8]. Information regarding the functional, positional and network properties of disease related genes are utilized by this method. Network measures like node connectivity and clustering coefficient values of molecular interaction networks have been extensively used to study the topological properties of disease genes [9,10]. Recently, it has been shown that disease genes avoid 'dense clustering neighborhoods' and have an average clustering coefficient of 0.015 [9]. Based on these properties, the positional candidates were first ranked by formulating a scoring method that assigned Weight value (Wv) to each candidate. Further, we exploited network measure to prioritize HWEs (candidates with a Wv > 0.5) referred to as HRC method (high weight elements ranked by clustering coefficient).

Comparison with other known prioritization methods which are based on sequence analysis as well as protein-protein interactions showed that our method had a gene enrichment ratio of 6.8 which is better than the other methods compared. We also predicted the functional processes brought about by positional candidates with special emphasis on those with unknown functions. This was done by constructing network motifs from the domain interaction network. These motifs represent specific biological functions and hence provide an insight regarding the physiological role of particular candidate. We believe such an approach which amalgamates functional, genetic and network properties could prove to be immensely helpful in ranking the positional candidates involved in disease.

## Results

Here, we integrated Wv method and domain interaction network property (HRC method) to predict positional candidates with a high likelihood to be involved in disease etiology. The calculated Wv for proteins ranged from 0-1 with the magnitude indicating relevance to T2D. The candidates with Wv > 0.5 were prioritized as high weight elements (HWEs). From the dataset of 4052 proteins, encoded by genes located within 10 Mb region encompassing 108 markers linked with T2D (LOD score ≥ 2), 995 candidates were classified as HWEs.

We assumed that most likely disease genes among HWEs should also share the network property. A recent study of network properties of disease genes has demonstrated that the disease genes avoid dense clustering neighborhood compared to essential genes and have an average clustering coefficient of 0.015 [9]. Hence we used average C_i _of 0.015 as a cut-off to prioritize HWEs. Ranking of HWEs based on their clustering coefficient (C_i _< 0.015) in the domain interaction network (HRC method) resulted in 435 most likely disease candidates termed as HWEc (Additional file 1). The topological features of the interactions derived here from domain interactions include an average degree of connectivity (k) of 6.5, an average clustering coefficient (C_i_) of 0.17 and the shortest path length of 4.5. Among the HWEs, 44% had C_i _< 0.015 (HWEc) whereas among the test dataset (refer methods for details) only 35% of the proteins had C_i _< 0.015 (p = 0.007). Our analysis revealed that majority of HWEc had less connectivity (k < 5; 47%) compared to test dataset which had preponderance of genes with higher connectivity (k > 5; 78%) (p < 1 × 10^-4^). This indicates that HWEc proteins have fewer links with low C_i _and might be less interconnected; these represent the network properties shared by disease genes. Furthermore, this also supports the assumption that genes that lie in the network-neighborhood of disease genes are more likely to be involved in disease causation. Using BiNGO [11] we assigned the biological processes from Gene Ontology (GO) shared by ranked candidates and found predominant involvement of HWEc in metabolic and signaling processes (Table 1).

**Table 1:** Gene ontologies revealed by BiNGO for ranked positional candidates

GO ID	Gene ontology - description	No. of ranked candidates	p-value
GO:0008151	Cellular process	279	1.3 × 10^-3^
GO:0008152	Metabolic process	237	1.8 × 10^-12^
GO:0044237	Primary metabolic process	225	3.6 × 10^-13^
GO:0044238	Cellular metabolic process	250	1.5 × 10^-13^
GO:0043170	Macromolecule metabolic process	198	1.7 × 10^-10^
GO:0019358	Protein metabolic process	160	1.2 × 10^-28^
GO:0044260	Cellular macromolecule metabolic process	159	2.0 × 10^-30^
GO:0044267	Cellular protein metabolic process	158	1.3 × 10^-30^
GO:0043283	Biopolymer metabolic process	144	4.1 × 10^-5^
GO:0065007	Biological regulation	138	4.3 × 10^-3^
GO:0050791	Regulation of biological process	118	0.018
GO:0051244	Regulation of cellular process	108	0.04
GO:0007154	Cell communication	106	0.026
GO:0006464	Protein modification process	100	4.9 × 10^-22^
GO:0043412	Biopolymer modification	100	5.4 × 10^-21^
GO:0007165	Signal transduction	97	0.036
GO:0043687	Post-translational protein modification	96	2.1 × 10^-25^
GO:0006796	Phosphate metabolic process	85	2.0 × 10^-30^
GO:0006793	Phosphorus metabolic process	85	5.0 × 10^-30^
GO:0016310	Phosphorylation	84	5.3 × 10^-36^
GO:0006468	Protein amino acid phosphorylation	83	2.3 × 10^-41^
GO:0051869	Response to stimulus	77	4.5 × 10^-3^
GO:0007242	Intracellular signaling cascade	55	2.2 × 10^-4^
GO:0006950	Response to stress	50	1.5 × 10^-7^
GO:0051242	Positive regulation of cellular process	38	7.5 × 10^-4^
GO:0002376	Immune system process	31	0.011
GO:0007243	Protein kinase cascade	27	0.017
GO:0006629	Lipid metabolic process	20	0.027
GO:0012501	Programmed cell death	27	0.034
GO:0006954	Inflammatory response	23	3.4 × 10^-6^

Top thirty biological processes enriched by 435 ranked positional candidates

### Functional relevance of network motif analysis

Network motifs that are the indicators of specific functional modules in cellular networks [12,13] were used to predict functions for hypothetical HWEc genes. For instance, network motif analysis showed that Q6MZN5 protein (Wv = 0.93, C_i _= 0.0) with domain PF05277 forms a four node motif with partners PF01565 (electron transport), PF02913 (electron transport), PF02127 (proteolysis) suggesting its role in energy metabolism. The predicted functions were also cross-validated using the Protfun server by matching with the functional classes assigned by Protfun. Protfun server also predicted the involvement of Q6MZN5 protein in energy metabolism. Energy generation in mitochondria occurs primarily through oxidative phophorylation and as genes involved in oxidative phosphorylation are known to be coordinately downregulated in T2D, Q6MZN5 might attain significance [14].

### Performance of the methods

**Figure 2:**
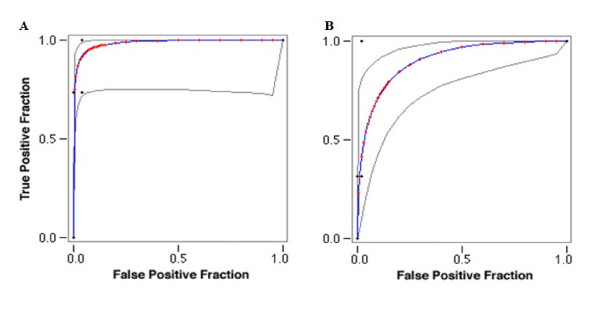
**Rank ROC curve obtained for disease validation**. (A) Weight value method. (B) HWEc method. Red symbols and Blue line: Fitted ROC curve. Gray lines: 95% confidence interval of the fitted ROC curve.

The benchmarking dataset used to evaluate the performance of our methods, comprised of 19 genes known to be associated with T2D from GWA studies [1,15] and 353 non-T2D genes lying within 10 Mb regions of 12 chromosomal positions those are never shown to be linked with any disease, as control data set. We observed sensitivity of Wv method to be 0.73 and specificity to be 0.96. For HRC method, sensitivity and specificity were 0.32 and 0.98 respectively. Overall data enrichment of 9 fold and 6.8 fold was observed for the two methods respectively. Using the benchmarking dataset, we compared both Wv and HRC predictions with other known methods of candidate gene prioritization (Table 2). The receiver operating characteristic (ROC) analysis of the two methods, Wv and HRC confirms their better performance (Fig. 2), with only PROSPECTR [16] having a higher sensitivity (0.90) than Wv and HRC. Where, G2D method gave accuracy and sensitivity of 0.73 and 0.53 respectively with the benchmarking dataset.

**Table 2:** Performance comparison of different methods using benchmarking dataset

	Wv	HRC	PROSPECTR	SUSPECTS	G2D	DGP
Accuracy (%)	94.9	**94.6**	41.4	61.1	72.8	49.8
Sensitivity (%)	73.7	31.6	**89.5**	52.6	52.6	58.8
Specificity (%)	96	**98**	38.8	61.5	73.9	49.3

Numbers in bold letters indicate significant values

Further, other gene prioritization methods including PROSPECTR [16], SUSPECTS [17], Disease Gene Prediction (DGP) [18] and G2D [19] were used to prioritize our dataset of 4052 genes. PROSPECTR and SUSPECTS gave a disease gene estimate of 1 in 1.7 (2275 in 3972 and 2254 in 3612 respectively), DGP did 1 in 2.1 (1720 in 3632) and for G2D it was 1 in 7 (524 in 4052). Whereas, Wv method gave disease gene estimate of 1 in 4 (995 in 4052), Wv + HRC provided an estimate of 1 in 9.31 (435 in 4052). Thus, it was striking that PROSPECTR, SUSPECTS and DGP predicted a higher number of genes to be disease candidates compared to our method. Among the other methods, G2D performed the best. While comparing the common candidates predicted by Wv + HRC and other methods we found that 84% (364) were prioritized to be disease candidate by any one of the methods while 16% (71) of the candidates were the unique candidates prioritized by our method (Additional file 2).

When compared with GeneWanderer (random walk) program [5] it was found that our method identified 6 disease genes compared to 7 by GeneWanderer (Table 3). Since, Wv approach includes prioritization based on the positional relevance of the candidate with relation to T2D linked regions and that some of the GWA genes do not lie in this region there is a likelihood of missing out on some candidates.

**Table 3:** Comparison with GeneWanderer (Random walk) method

Gene	Wv	HRC	Prioritized- Wv + HRC	Ranking by GeneWanderer Random walk method
*ADAMTS9*	**1**	**0**	**Y**	35
*NOTCH2*	**0.93**	**0**	**Y**	**9**
*HNF1B*	**0.87**	1		-
*PPARG*	**0.77**	0.667		**1**
*KCNQ1*	**0.73**	0.033		4
*CDKL1*	**0.63**	**0.005**	**Y**	16
*CAMK1D*	**0.63**	**0.005**	**Y**	30
*IGF2BP2*	**0.59**	**0.01**	**Y**	71
*LGR5*	**0.56**	0.286		41
*CDKN2B*	**0.53**	0.016		**1**
*CDKN2A*	**0.53**	0.016		**3**
*HHEX*	**0.53**	0.053		16
*TSPAN8*	**0.51**	**0**	**Y**	**10**
*TCF7L2*	0.48	0.03		-
*CDC123*	0.46	**0**		21
*MTNR1B*	0.44	**0**		**1**
*SLC30A8*	0.37	0.055		42
*HDAC2*	0.21	0.067		**9**
*KCNJ11*	0	**0**		-

The bold letters highlight significant values; Dataset comprised recently identified disease genes in T2D

### Prioritized Candidates by Wv + HRC method

Available literature suggests that candidates with high Wv and low clustering coefficient values, as prioritized by our method, show considerable relevance to T2D. For instance, the highest ranked candidate *GPC1 *(Wv-1.0, C_i_-0.0) is shown to influence FGF2 signaling pathway [20]. The angiogenic growth factor, FGF2 levels has been found to be associated with cardiovascular events in T2D [21]. *LPIN2 *and *LPIN3 *(Wv-0.98, C_i_-0.0) are the members of lipin family known to serve as an enzyme for triacylglycerol synthesis as well as transcriptional coactivator in the regulation of lipid metabolism genes [22]. *CDK5R2 *(Wv-0.98, C_i_-0.0) forms the complex with *CDK5*, a serine/threonine protein kinase that plays crucial role in physiological functions such as glucose stimulated insulin secretion in pancreatic cells [23]. *CX3CL1 *(Wv-0.97, C_i_-0.0), a CX3C chemokine has been shown to have specific role in initiation and progression of atherosclerotic vascular disease [24]. *VEGF *has already been shown to be associated with T2D complications [25]. *PDGFB *elevated levels and its induction by PKC activation has been shown to be involved in pathogenesis of diabetic retinopathy [26]. Mutation in *AKT2 *(R274H) enzyme has been shown to result in autosomal dominant inheritance of severe insulin resistance and T2D [27]. *CAMK2 *kinase found to be expressed in the pancreatic β-cell has been shown to affect insulin secretion [28]. Fetuin-A has been shown to be an important modulator of insulin resistance [29]. *CETP *plays an important role in the regulation of HDL metabolism and is shown to be associated with dyslipidemia in GWA studies [30]. *DMPK*, a serine/threonine protein kinase has been shown to be a positive modulator of insulin action [31]. *NDUFA5*, *NDUFS7*, *SCO1 *and *FMO4 *all are involved in oxidative phosphorylation and have been shown to be important in diabetes [14]. *MOGAT2 *was found to be associated with triacylglycerol synthesis and has a role in diet-induced obesity [32]. Interestingly, we found Bactericidal/permeability-increasing protein (BPI) as a highly ranked candidate and it has been suggested over production of *BPI *could be linked to insulin sensitivity and glucose tolerance [33]. A variation in Cathepsin S (*CTSS*), a cysteine protease has been shown to be associated human metabolic risk factors for cardiovascular diseases [34]. The other ranked candidate *PDE3A *in rat has been found to have antilipolytic action of insulin in adipocytes [35].

## Discussion

Identification and prioritization of the disease genes for complex diseases like T2D is inherently difficult. The etiology of T2D, though not very clear, involves multiple pathways wherein each probable disease gene confers only a modest risk. Thus, to understand the disease pathophysiology it is better to explore the global interaction network than single gene identities. These interactions if employed to prioritize genes might considerably increase the chance of detecting disease genes as perceived in our approach. The method relies on the disease-specific characteristics as reflected from the observation that 13 disease genes (Wv > 0.5) from total 19 (GWA studies in T2D) were identified. The performance of Wv and HRC methods showed accuracy and specificity better than other sequence based candidate gene prediction methods. The robustness of our method is well demonstrated by the benchmarking parameters. Furthermore, the GO sharing showed a higher representation of metabolic and signaling processes in the ranked genes. This confirms the common belief that genes associated with same disorder share similar functional characteristics [10,36]. Most of the ranked candidates were also found to be involved in diverse biological processes important in T2D like insulin secretion (CAMK2 kinase), HDL metabolism (*CETP*), modulation of insulin action (*DMPK*), oxidative phosphorylation (*NDUFA5*, *NDUFS7, SCO1 *and *FMO4*) and triacylglycerol synthesis (*MOGAT2*). Further, an immediate support of our work can be obtained from the recent association of *LPIN2 *gene to T2D [37]. Prioritization of candidates like *LPIN2 *as high ranked candidates, clearly indicate the efficiency and importance of our method.

As T2D is a polygenic disease involving multiple biological processes, it is imperative that disease genes will be rare in topologically central regions of network. It has been observed that the important functional modules are located in the dense regions of protein interaction network having high degree of connection and high clustering coefficient [9]. Also these dense regions of interaction networks probably perform the basic evolutionary processes with specialized functions being done by peripheral nodes [38]. Therefore, here we have used clustering coefficient (Ci < 0.015), in a domain interaction network to prioritize T2D disease candidates. This conglomerate modus operandi is evenhanded for genes encoding proteins having both known and unknown functions which are often ignored during disease gene identification.

The other known systems biology approaches for candidate gene prediction are based on direct protein-protein interaction of the gene that is being studied [4-6,39]. But, the limitation of these methods is that presently only 10% of all human protein-protein interactions have been described [40]. Here, we have tried to address this by exploiting the interactions of partner domains and their harboring proteins for prioritization. This increases the coverage of the search for the disease candidates in global interaction network. Notwithstanding this, a note of caution is warranted as the ranking of candidates here, could be affected by the appropriateness of the InterDom [41] and the score it provides, as the interaction networks are neither complete nor error free. Moreover, we have tried to encompass the true positive interactions by having the obstinate cut-off score, with validations from other data sources as well. Still, owing to the constraints in the gene annotation in the regions selected, availability of interaction data and that of ascertainment bias there is a possibility that a few plausible disease candidates would have missed out during the screening process.

## Conclusion

To achieve cost-effective experimentation and expediting the process of disease gene discovery it is essential to develop disease-specific methodologies rather than to rely solely on model-free approaches. Our method ranks the candidate genes in linked regions using T2D specific properties. We believe that performance of this method would improve with the availability of better gene and protein annotation and of true positive interaction data.

## Methods

The schematic representation of the methodology opted for the prioritization of disease gene candidates is presented in Fig. 1. Briefly, it involved the following steps and the detailed description of each step is provided below:

(i) Microsatellite markers with LOD scores ≥ 2.0 linked toT2D were selected (n = 108)

(ii) Candidates were retrieved from 10 Mb region encompassing each marker (5 Mb each upstream and downstream of the marker) from Ensembl and the protein sequences were extracted from SwissProt (n = 5441)

(iii) Domains were assigned for the proteins using pfam and their interacting partners were identified using InterDom. This resulted in a dataset of 4052 proteins.

(iv) Binary scoring was done for each partner domain of every candidate using the following classifiers a) domains present in proteins with T2D associated non-synonymous variations, b) domains that are involved in T2D related biological processes, c) domains present in proteins lying in T2D linked chromosomal regions d) domains in proteins associated with any other human disease as given in OMIM.

(v) Weight values (Wv) were obtained by analyzing the partners domains and candidates with Wv > 0.5 were called as High Weight Elements (HWEs)

(vi) HWEs with clustering coefficient value <0.015 in interaction network were called as HWEc,

(vii) Wv and network property of peripherality were integrated to prioritize the potential disease candidates.

### Selection of dataset

We searched the available literature for genome wide linkage scans in T2D and selected 108 microsatellite markers located on 64 different chromosomal regions with LOD scores ≥ 2.0 to select regions of profound statistical relevance with T2D (Additional file 3). All genes coding for known and unknown proteins within 5 Mb upstream and downstream of the selected markers (10 Mb region) were extracted from Ensembl database (v38) [42]. We ensured a non-redundant dataset comprising of 5441 candidate genes by screening for them in International Protein Index (IPI) [43].

### Domain assignment and prediction of partner proteins

The domain definitions used in this study were obtained from Pfam database [44]. The domains were assigned by scanning libraries of Hidden Markov models (HMMs) against the protein sequences resulting in a dataset of 4052 candidates which alone were considered for further analysis. Interacting partner domains for each parent domain (domains contained in the positional candidates) were determined using InterDom database with a confidence score of interactions ≥10 [41]. We utilized 26,058 domain interactions for Wv calculations from InterDom database (Version 2.0) after applying the cut-off of ≥10 for confidence score. These interactions were found to be evident either in DIP, BIND or PDB databases [45-47]. After obtaining the domain partners, all the proteins harboring them were extracted using SwissPfam [44].

### Calculation of Weight value using disease specific properties

Binary score of 1 or 0 was assigned to the partner domains on the basis of their presence or absence respectively in the following four classes:

#### **Class a**. *Domains present in proteins with T2D associated non-synonymous variations*

We used the dataset of proteins containing non-synonymous variations associated with T2D, derived from the previous study, as a scoring class [7]. The value of 1 or 0 was assigned based on the presence or absence of the domains in any of these proteins respectively. A total of 60 unique domains were identified to be present in the proteins implicated in T2D.

#### **Class b**. *Domains those are involved in T2D related biological processes*

Data from microarray-based studies in adipose tissue and skeletal muscle were used to determine the biological pathways involved in T2D [14,48]. We identified 15 such Gene Ontology (GO) biological processes and Pfam domains were manually categorized into these GO biological processes using Pfam2GO data (Additional file 4) [49]. The binary scoring was done based on the presence or absence of a partner domain in these GO biological processes.

#### **Class c**. *Domains present in proteins lying in T2D linked chromosomal regions*

A total of 64 chromosomal regions were identified as the location of the markers linked to T2D with a LOD score ≥ 2.0 (108 markers). Chromosomal locations for all the proteins containing the partner domains were obtained from Swiss-Prot database and were cross-checked with Ensembl. The score of 1 was assigned if proteins harboring the interacting domains were located on any of these linked chromosomal regions; otherwise value of 0 was assigned.

#### **Class d**. *Domains present in proteins associated with any other disease*

To determine the genes associated with human diseases, we searched entries in Online Mendelian Inheritance in Man (OMIM, release December 2005) database. A total of 1777 disease genes associated with 1284 disorders were found in OMIM. Redundant proteins falling under class 'a' category were eliminated. Proteins harboring the interacting domains were searched for the presence or absence in this dataset and values were assigned accordingly. Consideration of this category which incorporates the property of disease genes in the weight value scoring is of immense importance as T2D is a combination of many disorders.

### Weight Value (Wv) scoring

Let P_i _and P_j _be two interacting proteins where P_i _is the positional candidate that is being considered for prioritization and P_j _is the interacting partner. This interaction is assumed to be a result of interaction between their domains D_i _and D_j _respectively. Let D_i _be the parent domain and D_j _the partner, where j = 1, 2 ... N, as more than one domain can interact with D_i_.

The weight value for D_i _was calculated as follows:(1)

Where,

a_j _= D_ja_/T_a_

b_j _= D_jb_/T_b_

c_j _= P_jc_/T_c_

d_j _= P_jd_/T_d_

N = Total number of partner domains (D_j _interacting with parent domain D_i_)

Where,

D_ja _and D_jb _indicate the presence/absence of D_j _in classes a and b respectively.

P_jc _and P_jd _represent the presence/absence of P_j _in classes c and d respectively. Note that presence/absence is scored by a binary system.

However, it is more likely that more than one protein can contain D_j _and all the proteins harboring D_j _needs to be evaluated for categories c and d.

T_a_, T_b_, T_c _and T_d _are the total number of events in the classes 'a' through'd' which are 60, 15, 64 and 1746 respectively

Obtained Wv was normalized as follows:(2)

Subsequently, this Wv obtained for D_i _is allocated to P_i _as final outcome to rank the candidate genes dataset. For protein harboring more than one domain, highest Wv obtained amongst all the domains was assigned to the protein. Protein with Wv > 0.50 were called as HWEs.

### Ranking by clustering coefficient- HRC method

Proteins in HWEs were further ranked by determining network measure i.e. clustering coefficient (C_i_) in the domain interaction network (HRC). The clustering coefficient (C_i_) is 1 (high) when all the neighbors of a protein are linked to each other and small (C_i _= 0) if network is locally sparse [12]. Feldman et al reported that the two networks i.e. yeast two-hybrid (Y2H) and GeneWays (GW) used in their work are complementary rather than competing views of the human interactome [9]. Disease genes are known to avoid dense clustering neighborhoods and the average clustering coefficient for disease network observed was 0.015 [9]. Therefore, we have considered the lowest C_i _for the peripherality feature of the interaction network.

Therefore, HWEs were further ranked by using C_i _< 0.015 hypothesizing that those candidates below this C_i _could be probable disease candidates classifying them as HWEc. InterDom domain interactions (with a confidence score of interactions ≥10) were used for calculating the C_i _in NetworkAnalyzer [50].

### Benchmarking of prioritization

To assess the performance of Wv and HRC methodologies for prioritizing T2D related genes, we constructed a test dataset consisting: (i) already identified genes implicated in etiology of T2D by Genome wide association studies (n = 19) [1,15] and (ii) proteins encoded by genes located within 5 Mb region either side of 12 markers (n = 353) that are not linked to T2D (non-T2D genes). Further we analyzed this dataset by four other known independent sequence based methods viz. PROSPECTR [16], SUSPECTS [17], DGP [18] and G2D [19]. PROSPECTR uses sequence based features like gene length, protein length and percentage identity of homologs in other species and designates candidates as likely disease candidates if scored equal to or over a threshold of 0.5 [16]. SUSPECTS method is based on the annotation data from GO, InterDom and expression libraries [17]. Since, SUSPECTS does not define the cut off score for selecting disease genes, we have used a weighted-score of ≥10 based on the training dataset of genes used by Tiffin et al [3]. DGP assigns a probability score using a decision tree model based on sequence properties i.e. protein length, phylogenetic extent, degree of conservation and paralogy [18]. A probability score of ≥0.5 is assigned to all the disease proteins in the learning set used by DGP. Thus, candidates with a score of ≥0.5 were considered as prioritized disease genes in our dataset. G2D method scans a human genomic region for genes related to a particular disease based on the phenotype of the disorder or their similarity to an already known related gene [19]. The method performs BLASTX of the region against all the GO annotated in Refseq database and extracts putative genes in the chromosomal region and evaluates their possible relation to the disease. The default E-value (<10e^-10^) was used here for extracting the putative disease genes based on the "known gene" protocol of G2D. The "known gene" takes already well-known disease genes to be associated with disease of interest for prediction. Thus, here we have considered the same set of "known gene" in T2D used by the GeneWanderer program disease-gene families dataset [5]. As G2D takes chromosomal region for search, we have provided 10 Mb either side of 108 markers for searching putative genes. Measure of performance of methods was done by estimating receiver operating characteristic (ROC) curve, which plots true positive rate (TPR) versus false positive rate (FPR).

### Comparison with GeneWanderer

We also compared our prediction using 19 identified genes in T2D with recent GeneWanderer program. This approach ranks each candidate gene in a genomic interval (identified by linkage analysis) by their interactions to genes known to be involved in the phenotype/disease in the protein-protein interaction network [5]. Here, we have used the random walk method of GeneWanderer prioritization approach.

### Enrichment ratio

Enrichment ratio was estimated as the proportion of disease genes predicted by the methods divided by the disease genes within all benchmarking set as:(3)

Where ΣDG is all disease genes and ΣG is total number of genes considered here.

### Network analysis

#### Biological functions from interaction networks

Biological Networks Gene Ontology tool (BiNGO), a plugin of Cytoscape software [11] was used to determine which GO terms are significantly overrepresented in a set of high ranked candidates. We only selected the GO terms having ≥10 selected genes in particular biological functions. This analysis for biological functions was done for 391 from total of 435 genes.

#### Motif detection from domain interaction

The program 'mdraw' [51] was used to identify the network motifs from domain interaction network. The process of motif generation between real and randomized networks was a result of 1000 iterations. Motifs that recurred significantly (p ≤ 0.01) in the real networks compared to that of randomized networks were considered. Furthermore, the biological relevance of the network motifs was evaluated by annotating the Pfam domains to their respective biological processes. Functional classes derived from this analysis were subsequently verified using ProtFun 2.2 prediction server [52]. Protfun approach is based on sequence features like post-translational modifications and protein sorting for protein function prediction. Protfun server assigns functions to a protein by estimating the probability of it belonging to a functional class and odds values that sequence of entry belong to that class/category. The functional class with the highest score of probability and odds for each hypothetical candidate genes was considered.

## Abbreviations

T2D: Type 2 Diabetes; Wv: Weight Value; HWEs: High Weight Elements; HRC: High weight elements Ranked by Clustering coefficient; HWEc: High Weight Elements with clustering coefficient <0.015; GO: Gene Ontology

## Authors' contributions

AS and SC designed, processed, interpreted the data and wrote the manuscript. RT has contributed in work design and manuscript writing. DB has conceived and supervised the study. DB & NT have contributed by critical evaluation of the study and improving the manuscript. All the authors read and approved the final manuscript.

## Supplementary Material

Additional file 1 **Prioritized 435 positional candidates by Wv + HRC methods**. The file contains the list of the 435 ranked positional candidates with their clustering coefficients Click here for file

Additional file 2 **Number of overlaps and complementary genes identified by different methods in the total dataset (5441 candidates)**. The file details the total number of genes identified by four other prioritization methods and complementary set considering all the 5441 positional candidates Click here for file

Additional file 3 **T2D linked markers selected for the study**. The file contains the details of the 108 markers linked with T2D curated from literature considered for the study Click here for file

Additional file 4 **Biological processes shown to be important in T2D**. The file provides the information for the biological pathways involved in T2D from microarray based studies Click here for file
