# Supplementary material for: Gene prioritization in Type 2 Diabetes using domain interactions and network analysis
Source: BMC Genomics. 2010 Feb 2;11:84. doi: 10.1186/1471-2164-11-84 (PMC2824729; doi:10.1186/1471-2164-11-84)
Supplement: Additional file 2 — Number of overlaps and complementary genes identified by different methods in the total dataset (5441 candidates). The file details the total number of genes identified by four other prioritization methods and complementary set considering all the 5441 positional candidates [file 1471-2164-11-84-S2.DOC]

**Additional file 2**: Number of overlaps and complementary genes identified by different methods in the total dataset (5441 candidates)

|  | **G2D** | **DGP** | **PROSPECTR** | **SUSPECTS** | **Complementary genes** |
| --- | --- | --- | --- | --- | --- |
| **Wv+HRC** | 49 (11.5%) | 181 (42.6%) | 281 (66.1%) | 249(56.8%) | 71 (16%) |
| **G2D** |  | 174 (10%) | 186 (11%) | 186 (11%) | 338 (65%) |
| **DGP** |  |  | 1246 (66%) | 1187 (63%) | 382 (20%) |
| **PROSPECTR** |  |  |  | 1360 (61%) | 409 (16%) |
| **SUSPECTS** |  |  |  |  | 252 (11%) |

Numbers in each cell indicate the number of overlaps two corresponding methods. Percentages in the parenthesis indicate the proportion among the total genes identified by each method.
